# Competence in *Streptococcus pneumoniae* and Close Commensal Relatives: Mechanisms and Implications

**DOI:** 10.3389/fcimb.2019.00094

**Published:** 2019-04-03

**Authors:** Gabriela Salvadori, Roger Junges, Donald A. Morrison, Fernanda C. Petersen

**Affiliations:** ^1^Faculty of Dentistry, Institute of Oral Biology, University of Oslo, Oslo, Norway; ^2^Department of Biological Sciences, College of Liberal Arts and Sciences, University of Illinois at Chicago, Chicago, IL, United States

**Keywords:** streptococcus, competence, natural genetic transformation, CSP, *Streptococcus pneumoniae*

## Abstract

The mitis group of streptococci comprises species that are common colonizers of the naso-oral-pharyngeal tract of humans. *Streptococcus pneumoniae* and *Streptococcus mitis* are close relatives and share ~60–80% of orthologous genes, but still present striking differences in pathogenic potential toward the human host. *S. mitis* has long been recognized as a reservoir of antibiotic resistance genes for *S. pneumoniae*, as well as a source for capsule polysaccharide variation, leading to resistance and vaccine escape. Both species share the ability to become naturally competent, and in this context, competence-associated killing mechanisms such as fratricide are thought to play an important role in interspecies gene exchange. Here, we explore the general mechanism of natural genetic transformation in the two species and touch upon the fundamental clinical and evolutionary implications of sharing similar competence, fratricide mechanisms, and a large fraction of their genomic DNA.

## Introduction

Natural genetic transformation is a prime driver of evolution by promoting genetic variability through horizontal gene transfer (HGT) in both Gram-positive and Gram-negative bacteria. The ability to exchange genetic material via natural transformation is specially conserved across the genus Streptococcus, and is recognized to play an important role in the evolution of their genomes (Straume et al., 2015). During the last two decades, there has been significant increase in the understanding of the “big picture” of natural transformation processes in competent streptococci, with focus not only in physiological characterizations, but also at the gene regulation level. In addition, thanks to the rise of short and long sequencing read techniques, it is now possible to study evolution at the genomic level, leading to a better understanding of the impact of HGT and natural transformation in bacterial species (Cowley et al., 2018; Junges et al., 2018). Streptococci represent an extremely diverse group of more than 100 species, which colonize both humans and animals. Their interactions with the host, however, range from commensalistic to pathogenic relationships. In the mitis group, for instance, *Streptococcus pneumoniae* (the pneumococcus), asymptomatically inhabits the oral cavity and upper respiratory tract of humans. However, its migration to other regions is related to a wide variety of diseases, such as otitis media, pneumonia, meningitis, and septicemia (Kadioglu et al., 2008; Henriques-Normark and Tuomanen, 2013). On the other hand, other members of this group, such as *S. mitis, S. oralis*, and *S. pseudopneumoniae*, which colonize similar sites, generally behave as commensals, with restricted pathogenic potentials (Tappuni and Challacombe, 1993; Pearce et al., 1995; Wright et al., 2017). Evolutionary analyses suggest that these species share a common precursor with *S. pneumoniae*, and that they evolved from a pneumococcal-like ancestor by genome reduction, losing virulence genes and developing mechanisms to adapt to a commensal lifestyle with the shared human host (Kilian et al., 2014).

HGT is thought to play important roles in pneumococcal genetic variability related to virulence. Firstly, due to its high immunogenicity, the polysaccharide capsule is under strong selective pressure, which can lead to vaccine escape via natural genetic transformation as a means of genetic exchange of the complete polysaccharide synthesis loci (Kelly et al., 1994; Havarstein et al., 1997; Coffey et al., 1998). Secondly, through transformation, competent pneumococci can quickly integrate diverse antibiotic resistance genes and compromise the treatment of invasive pneumococcal disease, as for example by acquiring genes encoding variant penicillin binding proteins (PBP) from closely related species such as *S. mitis* and *S. oralis* (Hakenbeck et al., 2001; Chi et al., 2007). Interestingly, recombination events between *S. mitis, S. pneumoniae, S. oralis*, and *S. pseudopneumoniae* also comprise other genes associated with *S. pneumoniae* virulence and surface proteins. There is evidence of extensive recombination events within the cluster *S. pneumoniae*/*S. pseudopneumoniae*/*S. mitis* during their parallel evolution, which reflects their similarities. Puzzlingly, this exchange has been demonstrated mostly as a “one-way street,” with *S. mitis* and *S. oralis* as the DNA providers and *S. pneumoniae* as the recipient, even though a large proportion of *S. mitis* and *S. oralis* strains have the complete set of genes required for transformation (Havarstein et al., 1997; Kilian et al., 2008). Here, we briefly review the known mechanism of natural transformation in the mitis group and explore the evolutionary and clinical implications of the close relatedness and common features shared by *S. pneumoniae* and its nearby relatives.

## Horizontal Gene Transfer in *Streptococcus pneumoniae* and Close Relatives

Competence for natural transformation in streptococci is a temporary state, which in *S. pneumoniae* and other mitis streptococci can last up to 40 min (Håvarstein et al., 1995; Vickerman et al., 2007; Rodriguez et al., 2011; Salvadori et al., 2016). In this group, entrance into the competence state is triggered by a competence stimulating peptide (CSP); a hydrophobic pheromone produced and secreted by competent cells that elicits a response in sister cells in the same environment. This allows the cells to coordinate their entry into competence and differential regulation of genes involved in the uptake and recombination of extracellular naked DNA, as well as the production of killing factor against neighboring cells. The phenomenon of transformation was first observed *in vivo* by Griffith (1928), but later Dawson and Sia (1931) were able to reproduce the events *in vitro*, leading to the seminal experiments that ultimately led to the discovery of DNA as the hereditary material (Avery et al., 1944). Since then, competence has been studied mostly *in vitro*, in experiments that have revealed that recombinant DNA is imported into the cells as single strands of 2–3 kb up to ~6 kb (Fox and Allen, 1964; Lacks, 1966; Gurney and Fox, 1968; Morrison and Guild, 1973). Surprisingly, tracing the evolution of the PMEN-1 pneumococcal lineage isolated from different countries through 65 years, large segments of DNA of up to 30–50 kb in the capsule locus were identified as originating from other *S. pneumoniae* strains (Wyres et al., 2012). This could not be explained by conjugation or transduction alone, given the fact that there were not markers of conjugative plasmids, transposons, or mobile genetic elements in the vicinity of the acquired locus. Puzzlingly, however, the length of the acquired segments exceeded those usually attributed to exchanges mediated by natural transformation. Cowley et al. (2018) recently showed that cell-to-cell contact of competent and non-competent pneumococci in biofilms facilitates macrorecombination events of at least 30 kb, and that several transfers can happen simultaneously. Such large recombination events, interestingly, have also been observed in strains of *S. pneumoniae*, such as Hungary 19A, in which the donor DNA is apparently from the close relatives *S. mitis* and *S. oralis* (Kilian et al., 2008; Donati et al., 2010; Johnston et al., 2013), strongly suggesting that *S. pneumoniae* employs natural genetic transformation as means to acquire genes through HGT from closely related species, as well as within the species.

In *S. mitis*, there are still few clear indications for transfer of genes from *S. pneumoniae*. So far, analyses have revealed the occurrence of multiple homologous recombination events in different regions of the genomes of both species, such as gyrase and PBP, without clear traces of the direction of the transfers (Dowson et al., 1993; Coffey et al., 1998; Sauerbier et al., 2012). This is particularly interesting given the fact that these species are known to co-inhabit similar body regions, such as the human oral cavity and naso-oro-pharyngeal tracts, leaving them exposed to a favorable environment for gene exchange. Some possible explanations as to the apparent unidirectional gene flow from *S. mitis* to *S. pneumoniae* include a few hypotheses. Firstly, as *S. mitis* consists of a large group and includes several very distinct lineages, it is possible that current analyses with a restricted number of strains have simply not been extensive enough to pinpoint significant amounts of evident gene exchanges. If so, as more genome sequences for *S. mitis* become available, future phylogenetic analyses may allow identification of clearer recombination events involving pneumococcal donors and mitis recipients. Yet, *S. pneumoniae* has a variety of transposases and repetitive units (RUP) that are by far less represented in the genomes of *S. mitis* (Kilian et al., 2014). These are thought to contribute to the high genome plasticity of pneumococci. The second plausible explanation as for why there seems to be a “one-way street” in gene exchange would be the inability of some *S. mitis* strain to transform. Even though the majority of *S. mitis* possess the genes known to be essential for competence induction and maintenance, *sigX* appears to be truncated in ~1/3 of the strains that have been partially or completely sequenced (Kilian et al., 2014; Salvadori et al., 2016). Consequently, even though such strains possess the transformasome apparatus, transformation fails due to a lack of SigX-dependent regulation critical for DNA uptake and recombination. There is evidence, however, that transfer of homologous DNA seems to occur between *S. mitis* and *S. oralis* (Kilian et al., 2008). A recent report of *in vivo* interspecies gene transfer by Rieger et al. (2017) revealed the acquisition of a *pbp2x* mosaic gene of *S. pneumoniae* by a *S. mitis* strain, supporting a “two-way street” in gene exchange between the two species. Since the *pbp* gene is close to the capsule locus, recombination events in this region may also facilitate the transfer of capsule genes from one species to the other. Although this has been reported for *S. pneumoniae* strains, so far there are not indications that this has occurred between *S. mitis* and *S. pneumoniae*. It still remains unknown how frequently streptococci undergo genetic transformation in their natural human habitat. It is possible that for most of the time competence may be activated as a means to repair DNA, and that the signs of transformation *in vivo* that are observed by tracing back the acquisition of new genetic loci by selected isolates may represent rare occasions of incorporation of heterologous sequences.

One remaining gap in understanding transformation in *S. mitis* arises because competence under laboratory conditions has been notoriously difficult within this species (Gaustad, 1985; Bensing et al., 2001; Duran-Pinedo et al., 2014; Xie et al., 2015). However, recent findings show that by selecting appropriate growth media and using large PCR amplicon fragments as donor, *S. mitis* transformation can proceed *in vitro* at high levels (Salvadori et al., 2016). Such achievement opened for the possibility of further improving the transformation of encapsulated pneumococci, which even after the introduction of synthetic CSPs were known to transform with low efficiency *in vitro* (Ibrahim et al., 2004; Bättig and Mühlemann, 2008). This was in part attributed to the presence of the polysaccharide capsule that can reduce or block competence for DNA uptake. By employing the method developed for *S. mitis*, with minor modifications, *S. pneumoniae* D39 was shown to transform with efficiencies varying from 4 to 21%, similarly to *S. mitis* type strain, which is also encapsulated.

## The Competent State

In streptococci, the central regulation of the competence state, or “X-state,” depends upon a single master regulator, the alternative sigma factor SigX. Two main types of pheromone regulatory systems have been shown to control *sigX* expression in streptococci. The first is found in the mitis and anginosus groups, where *sigX* is regulated through a membrane-bound receptor of the TCSTS class, ComD, the cognate response regulator, ComE, and the CSP pheromone (Martin et al., 2006). The second type of signaling pathway occurs through a cytoplasmic receptor of the RRNPP class, ComR, and an imported small peptide pheromone, ComS, which has been found in species of the pyogenic, salivarius, bovis, and mutans groups (Gardan et al., 2009; Fontaine et al., 2010; Morrison et al., 2013). With the exception of *Streptococcus mutans*, which integrates the two systems to induce competence, each streptococcal species employs either a ComDE or a ComRS pathway. For some streptococci, like *S. pyogenes*, despite the presence of these regulatory systems, efforts to demonstrate competence in the laboratory have not fully succeeded (Mashburn-Warren et al., 2010; Marks et al., 2014).

**Figure 1:**
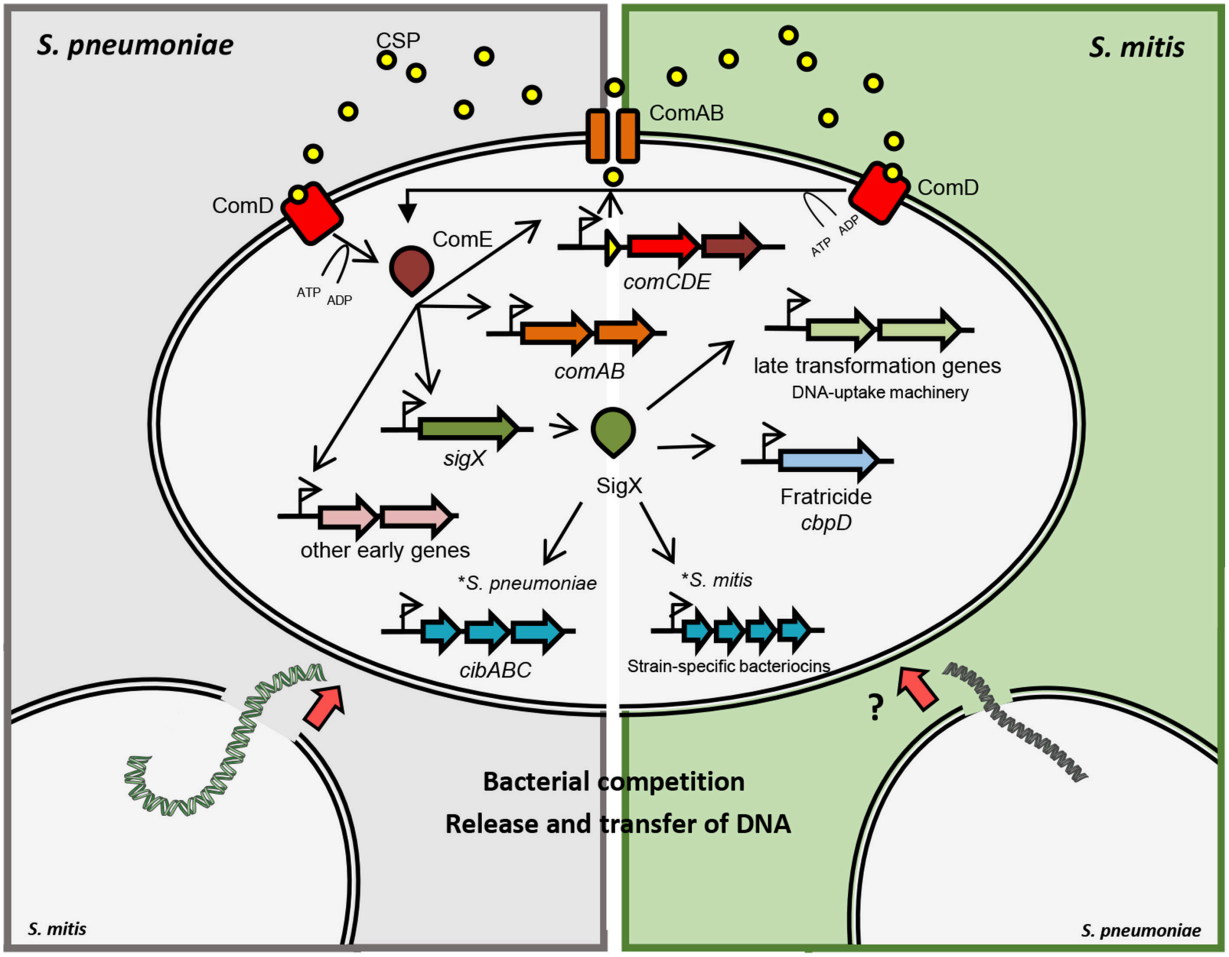
Schematic representation of competence gene regulation in *S. pneumoniae* and *S. mitis*. Pre-CSP is processed and exported by the ABC-transporter ComAB. CSP is detected by the histidine kinase ComD, which phosphorylates ComE. Phosphorylated ComE promotes the transcription of *comAB, comCDE* and *sigX*, in addition to other early genes. SigX regulates the expression of the DNA uptake and transformasome machineries, as well as the production of killing factors against other cells. In *S. pneumoniae* and *S. mitis*, fratricide depends on the late competence protein CbpD that lyses non-competent cells in the shared environment. Competent cells produce the early competence protein ComM, protecting themselves from CbpD lytic activity. Competent S*. pneumoniae* cells also produce CibAB, a two-peptide bacteriocin that acts on the membrane of non-competent cells. CibC is produced by competent cells in order to confer protection from the bacteriocin activity. Competent cells are then exposed to DNA released from the target cells that can be integrated into their genomes by homologous recombination. Competent *S. mitis* cells produce a different set of bacteriocins than *S. pneumoniae* under SigX regulation.

In *S. pneumoniae*, induction of competence is temporally divided into two stages (Figure 1). During the first 5 min after pheromone induction, there is the so-called early phase, controlled by the regulator ComE. In the mitis group, ComE regulates the expression of ~20 early competence genes, by recognizing and binding a specific motif in their promoter regions. The ComE regulon comprises the *comAB* and *comCDE* operons, as well as *sigX* (Lee and Morrison, 1999). ComE also controls the expression of *comW*, the product of which participates in SigX stabilization and subsequent activation (Luo et al., 2003; Sung and Morrison, 2005; Tovpeko and Morrison, 2014). The late CSP response is led by SigX, which bridges the early to the late genes, with an expression peak after 12 min of pheromone induction (Peterson et al., 2000, 2004). Although the mechanisms and conditions for acquiring competence may differ, the DNA uptake and recombination machinery forms, for its part, protein complexes conserved across species (Claverys et al., 2009).

## Finding the Core in the Middle of Chaos

Transcriptome analyses of competent streptococci have revealed that a range of 6–10% of the genome is upregulated in response to exogenous competence pheromones (Dagkessamanskaia et al., 2004; Peterson et al., 2004; Vickerman et al., 2007; Perry et al., 2009; Wenderska et al., 2017; Salvadori et al., 2018). This includes genes required for exogenous DNA uptake and recombination and genes involved in DNA scavenging after killing other closely related bacteria, but also a majority of genes not required for competence. Transcriptome comparisons across species reveal, however, a core SigX response in which two-thirds of the activated genes have already been shown to have a defined role in transformation, thus indicating that the system has evolved with the main purpose to enable streptococci to acquire DNA.

Streptococcal species have evolved from a common ancestor, mastering abilities to survive in different hosts and environments, by means of mutations, gene shuffling between different genomes, and gene gains and losses (Richards et al., 2014). Remarkably, competence for genetic transformation has been maintained as a trait of all streptococcal species, is universally dependent on SigX (Johnston et al., 2014), and relies on a conserved set of genes under SigX regulation. Recent data gathered from several transcriptome analyses of competent streptococcal species revealed a “signature” of SigX regulation, with a core of 27 to 30 pan genes, 20 of which are involved in binding, uptake, processing and integration of recombinant DNA, in addition to the production of killing factors against other cells (Khan et al., 2016). Interestingly, comparison of transcriptomes in different strains of competent *S. mitis* has revealed that bacteriocin genes are strain specific and are under SigX regulation (Salvadori et al., 2018). This highlights important differences between *S. mitis* and *S. pneumoniae* that have the potential to influence the DNA source available for transformation, and influence their abilities to compete for host colonization.

## Fratricide and Its Implications

In parallel with gene regulation required for genetic transformation, SigX controls a competence-induced cell lysis mechanism, known as fratricide. Through fratricide, competent cells cause lysis of non-competent cells in the same environment, releasing their DNA presumably for transformation (Steinmoen et al., 2003), but also contributing for biofilm formation (Petersen et al., 2004). This process has been proposed as a predatory mechanism that also contributes to pathogenicity by regulating the release of different virulence factors such as pneumolysin (Claverys and Håvarstein, 2007). Fratricide has been thoroughly studied in *S. pneumoniae*, and the murein hydrolase choline-binding protein D (CbpD) together with the “early” immunity protein ComM were identified as the key players in the process of lysing the target cells (Kausmally et al., 2005; Eldholm et al., 2009). This phenomenon was initially observed and described in mono-specific cultures and was initially shown to facilitate gene transfer between strains of pneumococci. However, fratricide was extended to an inter-species affair with broader ecological significance when Johnsborg et al. (2008) demonstrated that the pneumococcal CbpD could also elicit release of DNA from closely related species, such as *S. oralis* and *S. mitis*. Indeed, CbpD was later shown to act by binding to choline-decorated teichoic acids, which limits its cross-species activity to those who have choline in their cell wall such as *S. mitis* and *S. oralis* (Eldholm et al., 2010). From such perspective, being exposed to a highly selected and larger gene pool from lysed close relatives provides *S. pneumoniae*, and arguably, their close relatives *S. mitis, S. oralis*, and *S. pseudopneumoniae* with a favorable environment for exchanging genetic features within their own group.

Small peptide bacteriocins mediate antagonist activities in different bacterial species sharing the same ecological niche and favors competition between commensals and pathogens ultimately leading to successful colonization (Wang and Kuramitsu, 2005). After decades of studies on competence for genetic transformation in streptococci, it is well-established that the state of competence is tightly linked to the production of bacteriocins, through varied regulatory pathways (recently reviewed by Shanker and Federle (2017). Lysis of non-competent pneumococcal cells in the same environment does not rely only on CbpD, as the two-peptide bacteriocin CibAB also participates in the process of allolysis (Guiral et al., 2005). In other species of the mitis group, however, the pair of bacteriocins CibAB does not seem to be conserved, and various species-specific bacteriocin genes have been identified. In recent transcriptome analysis of competent *S. mitis*, two strain-specific operons under SigX regulation coded for different two-peptide bacteriocins without orthologs in other streptococcal species; these are also likely involved in competition with other cells and possibly as a mechanism to scavenge DNA (Salvadori et al., 2018; Figure 1). Notably, the presence of genes putatively involved in the production of immunity to these bacteriocins were also identified in both strains. These findings indicate that the competence system in the mitis group of streptococci, when functional, orchestrates the production of bacteriocins in a species-specific and, in *S. mitis*, a strain-dependent manner under SigX regulation. Altogether, the knowledge about the regulation of bacteriocins and its intertwined connection with competence may explain the importance of their parallel evolution and their role in predatory mechanisms within the mitis group of streptococci.

## The Puzzle of ComC Polymorphisms in the Mitis Group

**Figure 2:**
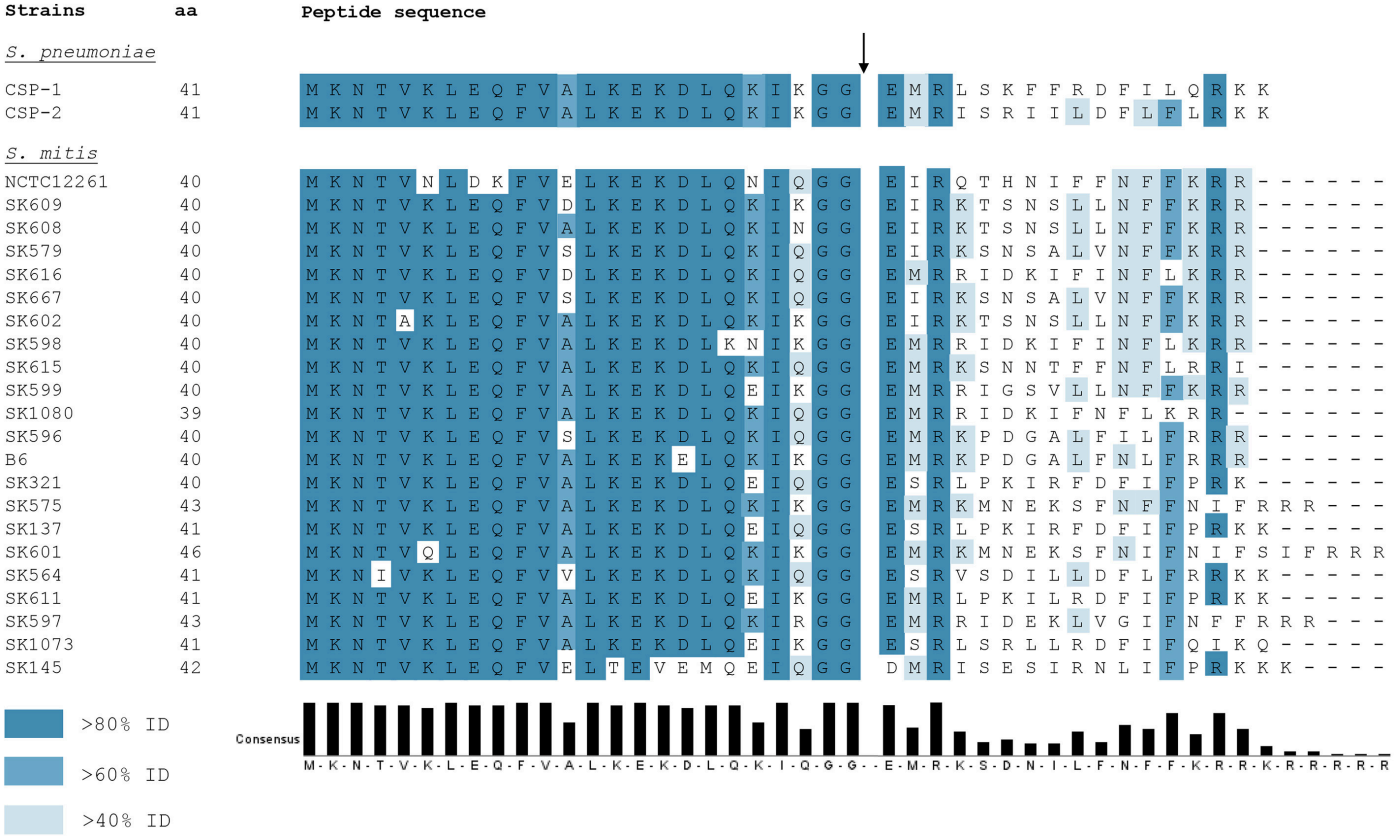
ComC polymorphism among 22 *S. mitis* strains and *S. pneumoniae* CSP-1 and CSP-2. Blue colors the residues according to the percentage of the residues in each column that agree with the consensus sequence. Only the residues that agree with the consensus residue for each column are colored. The black arrow indicates the CSP processing site. Full CSP sequences for available strains were obtained from Kilian et al. (2008).

Ever since the identification and characterization of *S. pneumoniae* CSP by Håvarstein et al. (1995), other CSP variants in mitis group streptococci were identified. Differently from *S. pneumoniae*, in which most of the strains encode one of two allelic variants—CSP-1 or CSP-2 (Pozzi et al., 1996) -, *S. mitis* presents extensive polymorphism in the *comC* gene within the species (Kilian et al., 2008; Figure 2). Such diversity in active CSP sequences presents a scenario where, for instance, several strains of *S. mitis* belonging to different pherogroups (bacteria with identical or cross-reactive pheromones) might possibly be interacting in the natural environment. From a physiological perspective, communication between *S. mitis* strains may be restricted by the specificity of the CSPs they produce. Hence, different strains might co-exist in multispecies biofilms in the oral cavity and nasopharynx without being able to communicate with each other via CSP. Still, belonging to different pherotypes increases the likelihood that competent *S. mitis* cells attack and acquire DNA from each other via the attack by the competence-induced bacteriolysin CbpD (Claverys and Håvarstein, 2007; Claverys et al., 2007). In a scenario where the co-infecting *S. mitis* strains present the same pheromone specificity, trading genes may be reduced, since each strain would raise its defenses when the other becomes competent. By having many pherotypes, almost all pairings would be hetero-specific, allowing inter-strain fratricide and gene exchange, despite each having protection from self-attack *via* ComM. Indeed, *S. mitis* strains are known to be unusually diverse. Cross-reactivity between pheromones in the mitis group has been shown only in *S. pneumoniae* R6, in which a promiscuous ComD receptor is able to detect CSPs produced by two *S. mitis* strains (type strain and SK612), and readily elicits its defenses through transcription of *comM* (Johnsborg et al., 2008). This may represent a beneficial asset to strains sharing the same habitat with others from different pherogroups. Although no exploratory studies on this subject have been done in *S. mitis* populations, a recent study on pherotype polymorphism in *S. pneumoniae* documented significantly higher rates of genetic exchange between strains of different pherotypes than among isolates presenting the same pherotype (Miller et al., 2017). From such a perspective, being exposed to a more diverse genetic pool might also increase the likelihood of acquiring new characteristics, thus increasing genetic variability.

## Future Perspectives

Genetic exchange *via* natural genetic transformation may help streptococci to adapt to the constant environmental changes in the oral and upper respiratory tracts. Over the past two decades, many studies have elucidated and described the mechanisms and genes behind competence regulation that led to a better understanding of the impact of natural transformation in *S. pneumoniae*. This is in contrast with *S. mitis*, a commensal relative that only recently has proven to transform at levels that enable using genetic tools to unravel its biology (Salvadori et al., 2016). The lack of focus on commensals still prevails in research, and is illustrated by the <100 *S. mitis* genome sequences available, in contrast with the more than 8,000 of *S. pneumoniae*. Yet, sequences of *S. mitis* and other close relatives are already providing an answer to the long-standing mystery as to the source of the variable serotypes in *S. pneumoniae* (Kilian et al., 2014; Lessa et al., 2018; Pimenta et al., 2018). It will be exciting to see whether future studies that consider the diversity in *S. mitis* strains will further reveal important information for our understanding of pneumococcal genome plasticity and pathogenicity.

## Author Contributions

GS, RJ, DM, and FP conceptualized the manuscript, drafted, and critically revised the manuscript. All authors approved the final version of the manuscript for publication.

### Conflict of Interest Statement

The authors declare that the research was conducted in the absence of any commercial or financial relationships that could be construed as a potential conflict of interest.
